# Structural Exploration and Conformational Transitions in MDM2 upon DHFR Interaction from *Homo sapiens*: A Computational Outlook for Malignancy via Epigenetic Disruption

**DOI:** 10.1155/2016/9420692

**Published:** 2016-04-17

**Authors:** Arundhati Banerjee, Sujay Ray

**Affiliations:** ^1^Department of Biotechnology, National Institute of Technology, Mahatma Gandhi Avenue, Durgapur, West Bengal 713209, India; ^2^Department of Biochemistry and Biophysics, University of Kalyani, Kalyani, Nadia, West Bengal 741235, India

## Abstract

Structural basis for exploration into MDM2 and MDM2-DHFR interaction plays a vital role in analyzing the obstruction in folate metabolism, nonsynthesis of purines, and further epigenetic regulation in* Homo sapiens*. Therefore, it leads to suppression of normal cellular behavior and malignancy. This has been earlier documented via yeast two-hybrid assays. So, with a novel outlook, this study explores the molecular level demonstration of the best satisfactory MDM2 model selection after performing manifold modeling techniques. *Z*-scores and other stereochemical features were estimated for comparison. Further, protein-protein docking was executed with MDM2 and the experimentally validated X-ray crystallographic DHFR. Residual disclosure from the best suited simulated protein complex disclosed 18 side chain and 3 ionic interactions to strongly accommodate MDM2 protein into the pocket-like zone in DHFR due to the positive environment by charged residues. Lysine residues from MDM2 played a predominant role. Moreover, evaluation from varied energy calculations, folding rate, and net area for solvent accessibility implied the active participation of MDM2 with DHFR. Fascinatingly, conformational transitions from coils to helices and *β*-sheets after interaction with DHFR affirm the conformational strength and firmer interaction of human MDM2-DHFR. Therefore, this probe instigates near-future clinical research and interactive computational investigations with mutations.

## 1. Introduction

In the proper and proficient cytological and neurological performances, a pivotal role is played by the tumor suppressor protein for executing an eminent responsibility for cellular regulations and repairing of DNA in humans [1]. To inhibit and suppress the cellular proliferation during oncogenesis, p53 actively participates in the process and thereby shields the host cell from malignancy [2].

An E3 ubiquitin-protein ligase MDM2 (mouse double minute 2) is a well-known protein possessing ubiquitin ligase activity in humans [3, 4]. In* Homo sapiens*,* MDM2* gene encodes this ubiquitin ligase protein [3, 4]. This protein from human interacts with p53 directly to inhibit the efficient functioning of p53 protein in malignant cells [5, 6]. Along with that, in a p53 independent manner, MDM2 was observed to enhance the degradation of varied proteins indulged in the process for proper cellular growth and progression of human cytological development [7]. One of such paramount proteins to which human MDM2 binds in* Homo sapiens* is DHFR (dihydrofolate reductase) [7]. DHFR or dihydrofolate reductase is a distinguished enzyme that converts dihydrofolic acid, with the aid of NADPH (electron donor), to tetrahydrofolic acid [8, 9].* DHFR* gene in humans encodes the responsible prominent protein: DHFR [8, 9]. DHFR protein holds a prime functionality in the synthesis of purines and thymidylate in humans [10]. These retain a vital responsibility in cell proliferation [10]. DHFR also performs a distinct central responsibility in the generation of precursors for adenine, guanine, thymine, uracil, and cytosine [10].

Earlier biochemical wet laboratory experimentations were performed in this regard through preparation of cultures, transfection, preparation of columns, mass spectroscopy, Western-Blot analysis, and* in vitro* binding assays, sequentially [7]. These finally revealed MDM2 to be a DHFR-binding protein. Moreover, to bring the results in accord with the data analyzed, yeast two-hybrid assays also confirmed that MDM2 utilizes this DHFR protein as its substrate and thereby interacts with DHFR to perform its only one approach of ubiquitination, monoubiquitination [7]. Therefore, it minimizes and disables the performance of DHFR protein without the interference of p53 protein [7]. This hampers the metabolism of the folic acids in human cells [7]. In the process, methylation of important proteins and nucleic acids and necessary metabolism of DNA get hampered leading to abnormal cell growth and proliferation of malignant cells [7].

So far, several wet laboratory researches have been documented in this regard for the activity of MDM2 protein for G1 cell cycle arrest [11] as well as interaction of MDM2 and DHFR protein [7], but, to date, no molecular level or computational study has been undertaken to investigate the switches and alterations in the MDM2 conformation and its structural description upon optimization, simulation, and interaction of DHFR. So, this present study endeavored to primarily model the human MDM2 protein in varied distinct molecular modeling techniques and approaches. The most suitable, satisfactory, and validated model was chosen for the further study after a comparable investigation among all the modeled structures. The selected model of human MDM2 was then optimized and simulated to bring its conformation closer to its native structure. Human DHFR was analyzed from the experimentally validated X-ray crystal structure. The modeled best suited MDM2 protein was then made to interact with human DHFR protein via protein-protein docking phenomena. The residual disclosures and the contribution of the responsible residues from the individual proteins were explored. The conformational transition in MDM2 protein before and after interaction was analyzed. The free energy of folding, folding rate of the protein for the thermodynamic stability of the protein, and the net solvent accessible area for the paramount and essential residues from MDM2 wereinvestigated and compared with their respective values before interaction. The outcomes were evaluated to be statistically significant too.

Formerly, several such studies involved the molecular stage disclosures of varied proteins responsible for human diseases and human cell cytology but, till present time, none included MDM2 and DHFR and their relationship for the epigenetic progress [12–14]. Therefore, this study with a computational and simulation approach examines the detailed molecular level involvement into the structural and residual contribution of MDM2 protein and DHFR protein from humans. Thus, it draws a link between MDM2, significant activity of ubiquitin ligase, and an imperative pathway for one-carbon donor engrossed in epigenetic regulation and metabolism of DNA. This holds a novel approach for cancer research and epigenetic metabolism and holds distinct insinuations for development of tumor and cytology.

## 2. Material and Methods

### 2.1. Analysis of the MDM2 Protein Sequence and Exploration into Templates

Amino acid sequence of the MDM2 protein is the main essentiality to be extracted for the purpose of modeling the protein. The protein sequence was obtained from NCBI with a proper validation from UniProt. MDM2 protein sequence from* Homo sapiens* possessed an accession number Q00987. The sequence was subjected to PSI-BLAST [15] to search for its potential templates against the PDB. It helps to predict the sequences that are far remotely related also. The search for human MDM2 protein sequence led to the template having PDB ID 4XXB with its B chain from* Homo sapiens*. The query coverage and the sequence identity of the target MDM2 sequence and the template sequence were 30% and 100%, respectively. Previous studies state that if the criteria for sequence identity between the target and template sequence serve to be at least 30%, then the target protein can be homology modeled [16]. In this present investigation, although the sequence identity showed 100% but the query coverage between the most proficient X-ray crystal template (PDB ID: 4XXB_B) and the human MDM2 sequence yielded only 30%, manifold modeling approaches were attempted to select the best possible validated 3D model for MDM2 protein.

### 2.2. Discrete Molecular Modeling Techniques for MDM2 Protein from* Homo sapiens*

#### 2.2.1. Homology (Comparative) Modeling of MDM2 Protein

With the operation and specific commands in MODELLER 9.14, the homology model of the respective MDM2 protein was built [17]. It is well known that minimal errors in the side chain packaging and loop conformations occur for the modeled protein, when the required sequence identity is more than 50% [18]. To model and eradicate the errors (if any) in the loop conformations for the MDM2 protein, MODELLER performs loop optimization and thus remodeling of the entire protein model. Therefore, it brings about appropriateness in *ϕ*-*φ* angles of the modeled protein [17].

#### 2.2.2. Remote Homology Modeling of MDM2 Protein

One of the most exigent aspects is held by the remote homology modeling from the sequence of a protein. The reason serves to be that, in comparison to the amino acid sequence of the target protein to be modeled, the structure of a protein remains more conserved by nature throughout the lengthy evolutionary pathways [19]. To perform and efficiently execute the remote homology modeling of MDM2 protein, Raptor-X [19] was operated. It is distinguishable for aligning tough target protein having sequence identity even less than 30% and holding a solved structure in PDB (Protein Data Bank) [19].

#### 2.2.3. Two-Way Approach for Fold Recognition Technique for MDM2 Protein

I-TASSER [20] as well as Phyre2 [21] was operated for the two-way approach in the threading technique (fold recognition technique) for modeling the MDM2 protein from humans. I-TASSER takes into consideration a particular prototype that considers sequence, structure, and function of the protein model in a sequential manner [20]. It therefore performs the modeling of the 3D protein structure preserving its functionality [20]. The utilization of the algorithm that utilizes profile-to-profile match was considered while operating Phyre2 [21]. This makes it unique amongst all such 3D structure prediction algorithms [21]. It can thereby be consistently distinguished twice as many homologues that are remotely related [21].

### 2.3. Comparative Evaluations for the Best Modeled Protein Selection

Certain stereochemical properties of the modeled MDM2 proteins were validated competently. To estimate the *Z*-score values of the individual MDM2 modeled protein, ProSA was operated [22]. To evaluate the alteration of the net total energy of the individual MDM2 modeled structures with respect to the assortment of their energy values from abrupt conformations of the respective protein, *Z*-score value is computed [22]. The more the negativity in the *Z*-score value for a modeled protein (here, MDM2), the closer the structure to its native form with improved appropriateness [22]. The overall stereochemical features including the main chain characteristics of the four distinctly modeled MDM2 protein structures were substantiated by SAVES server. Verify3D was evaluated and compared for each and every modeled structure to determine the conviviality of all the modeled MDM2 protein with relevance to its own amino acid sequence and therefore the best suited value was perceived [23]. To observe the allotment of residues (from the individual modeled proteins) into the core (most favored) and disallowed regions in the Ramachandran plots, such relevant Ramachandran plots [24] were mapped.

### 2.4. Proper Conformation of the Loop Regions in MDM2 Protein

Even after efficient modeling of a protein, several structural disparities involving deformities in the loop regions and errors in the packaging of the side chain atoms might occur [25, 26]. Such loop regions are known to reside in the disallowed or slightly favored regions in the Ramachandran plot. So, for a better improved conformation, these loop regions need to get optimized and remodeled. For this purpose, ModLoop was operated with the aid of MODELLER to build the optimized structure of the best suited MDM2 protein [27]. For loop optimization, the locations of every non-hydrogen-bonded atom in the loop are optimized and minimized in a certain environment with reference to an ersatz energy function [27]. The calculated energy value is the total of the restraints in the spatial atoms involving bond angles, length of the bonds, and the inappropriate dihedral angle via CHARMM-22 force field [27]. Furthermore, these values add to the statistical predilections for the dihedral angles and atomic interconnections that are nonbonded in nature [27]. The atomic connectivity that is nonbonded in nature depends upon dual atomic nature, which is their separation in their respective space and in their sequence [27]. With the usage of MODELLER, the optimization of the energy function is carried out with the utilization of conjugate gradient method accompanied by molecular dynamics plus simulated annealing [27]. Experimentally, it was earlier observed that the calculated loop conformation corresponds to the minimum conformation in energy amongst five hundred nonreliant optimizations. In the experiment, calculations were performed for forty loops of validated structures possessing length of 1–14 residues each [27]. The precision in envisaging the loop conformations was estimated as a function of meticulousness of the sampling of the loop conformations, length of the individual loops, and the structural characteristics of the native loops. In a similar pattern, the improved conformation for the specific *ψ*-*φ* angles in the selected MDM2 is obtained [27]. Additionally, the proper conformation of the modeled MDM2 protein is obtained with the satisfaction of the spatial controls [27].

### 2.5. Protein Model Optimization for Energy Refinement

This loop optimized MDM2 protein further is improved to attain a stable and refined final conformation with the performance of energy optimization and refinement. For this purpose, ModRefiner was operated [28]. An algorithm was, therefore, operated to begin the energy refinement process from the basic atomic model considering C_*α*_ atoms [28]. High resolution protein structure for MDM2 was obtained. A combination of dual force field aided the process [28]. The two force fields were template information and physics dependent [28]. This leads to the formation of the whole-atom 3D conformation in a protein. Two essential criteria are taken into consideration for preparation of atomic refined models [28]. The first one serves to calculate the topological identity of the modeled protein to the experimental structure, in a global approach [28]. It includes the evaluation of root mean-square deviation (RMSD), first. Then, further, for the purpose of calculation of accuracies in the backbone atom conformations to resemble the native structure, template modeling (TM) score is estimated [28]. Additionally, to evaluate the relationship between the global atoms, the “global distance test-total score (GDT-TS)” is also evaluated [28]. The second criterion lies in the physical pragmatism of the detailed atomic conformations. In addition to that, qualities based on local structural conformations, involving bond angles, bond length, steric clashes, side chain conformations, and torsion angles, are observed so that they resemble the features perceived in the native experimental structures [28].

The final modeled MDM2 protein was thereby brought very close to its native state form. This is because at this specific stage the structure tends to be more interactive and thereby most stable [28].

### 2.6. Structure Extraction of DHFR Protein from* Homo sapiens*

For the present study, to analyze the 3D functional structure of DHFR protein from* Homo sapiens*, an experimentally validated X-ray crystallographic DHFR functional structure was perceived from PDB [29]. The structure was in interaction with NADP+ and folate for its normal folate metabolism [29]. The deposited protein also underwent molecular dynamics simulations efficiently, as per the documentation from the research study [29]. The PDB ID for the DHFR protein was 4M6K with chain A [29]. Chain A protein from the protein-ligand complex in 4M6K was extracted using Discovery Studio packages from Accelrys. The so-obtained protein DHFR was thereafter observed to possess 186 amino acid residues in total.

### 2.7. Protein-Protein Docking for MDM2-DHFR Complex

To perform the interactive study between MDM2 and DHFR proteins, the proteins were docked amongst themselves via the performance of protein-protein docking with the operation of Cluspro2.0 [30]. The protein being shorter in length was provided as ligand, whereas the other partner participated as receptor. The unstructured residues from both partner proteins were omitted with the operation of the advanced structure amendment option in Cluspro2.0 [30]. A total of 10 distinctive complex protein structures were obtained as output after the efficient clustering followed by the overall minimization of the complex structures. Mainly, dependence upon the most apt and steady evaluation for the calculation of the desolvation free energy, as well as electrostatic energy (ahead of clusterization), the most pertinent protein complex structure for DHFR and MDM2, was selected. The output serves as a rank-wise record for the protein-protein complexes. The complex structure (ranked as model zero) with the best suited cluster and the best interactive form was opted for for further analysis.

For obtaining consensus and comprehensive outcomes for the protein-protein docking complex, Z-DOCK [31] and GRAMM-X [32] were operated too. In the both cases, scoring functions were calculated. The scoring function in Z-DOCK is based upon energy by nature, whereas the other one (from GRAMM-X) is knowledge dependent by nature [31, 32]. Additionally, Z-DOCK also considers the rotational gaps between the proteins, besides considering the translational gaps [31]. Both algorithms utilized fast Fourier transformation for the docking mechanism [31, 32]. Subsequently, the complementarities for the respective protein contour and energy estimations like desolvation free energy as well as electrostatic potential energy were once again estimated while utilizing the algorithm in Z-DOCK [31]. Linux cluster (320 processors) helped in the docking programs also [31, 32]. The best DHFR-MDM2 complex model from each of the operations was chosen, once again from the respective most steady energy estimations.

### 2.8. Molecular Dynamics Simulations for the Selected MDM2-DHFR Complex

Molecular dynamics simulations are necessary to obtain a stable and interactive conformation for the selected DHFR-MDM2 protein complex. For this purpose, FG-MD (Fragment Guided Molecular Dynamics) simulation was operated [33]. AMBER99 force field was utilized to perform the optimization of the DHFR-MDM2 protein complex after the additional optimization of the H-bonds [33]. After steady optimization of the complex, molecular dynamics simulations were performed with the concurrent application of physics and knowledge dependent template information as well as repugnant potentials [33]. Primarily, in the process, the entire protein complex model is fragmented into several segments. After that, the fragmented segments are assembled together to obtain the final stable DHFR-MDM2 complex structure [33]. Following the simulated annealing phenomena in molecular dynamics, the pathway for the overall energy was reprepared [33]. With the additional improvement of the torsion angles, the steric clashes were eradicated too. In the due process, the final simulated complex structure for DHFR-MDM2 nears to its native conformation with an elevated stage of accuracy [33]. Therefore, the final refined and simulated DHFR-MDM2 complex structure was obtained.

### 2.9. Interactive Residues and Binding Modes in the DHFR-MDM2 Protein Complex

The stable, refined, simulated, and final DHFR-MDM2 protein complex structure was considered for the study of the residual participation from the individual distinct proteins. For this purpose, the interactions as well as the binding patterns were observed by the operation of Binding Site Analysis tool in Discovery Studio packages from Accelrys as well as Protein Interaction Calculator (PIC) [34]. Disulphide bonds, ionic interactions, aromatic interactions, cation-*π* interactions, aromatic-sulphur interactions, and hydrophobic and hydrogen interactions were investigated [34]. Ionic interactions are documented from previous investigations to be the most strengthening bonds in a protein complex [35].

### 2.10. Conformational Transitions in MDM2 before and after Interaction

The switches in the conformation of the MDM2 protein in its preinteractive stage and, on the other hand, in its simulated interactive state with DHFR were evaluated. Documentation suggests that as the percentage of residues adopting helical and sheet-like conformation increases with the reduction in the percentage of the coil-like conformation, the protein tends to attain stable and steady conformation [36]. Therefore, the conformational transitions in MDM2 were observed with the execution of DSSP method [37], as well as Discovery Studio packages of Accelrys. Both provided the same outcomes. Additionally, assistance of PyMOL [38] helped to authenticate the upshots further.

### 2.11. Evaluation for the Strength and Stability of Interaction with DHFR

#### 2.11.1. Energy Evaluations for MDM2 Protein before and after DHFR Interaction

We estimate the strength and steady interactive nature of the MDM2 after interaction with DHFR protein which was evaluated from its free energy of folding values and DFire energy calculations [39, 40]. The interatomic interaction and firm participation of the MDM2 and DHFR proteins were evaluated from DFire energy calculations [40]. Free energy of folding value (in terms of Δ*G*) helps to infer about the foldability of the MDM2-DHFR protein complex after interaction [39]. Earlier experimental results suggest that the nonbonded interactions amongst the atoms of the modeled protein structure were estimated by DFire estimations [40]. The protein interactions are estimated with respect to their exact geometric conformation [40]. It is performed by taking into account the polar atoms to behave as a dipole and thereafter to have a direction. Therefore, it takes into consideration and focuses upon the dipole-dipole interactions [40]. This leads to the calculation of statistical energy function via the examination of the separation and angle reliance for the individual respective atoms [40]. Therefore, with the analysis of the accurate restoration of the side chain and main chain conformations, the best suited conformation of the protein structure near to its native state was selected. Thus, the protein having the least DFire energy value affirms having a native-like conformation and thereby is more stable in nature [40]. An ascent in Δ*G* value surmises an improved and ordered functional structure of the individual protein with a better foldability in the protein [39, 40]. The thermodynamic stability and the adaptation of the protein to attain an ordered structure with better folds in the protein were therefore estimated via free energy of folding [39]. This leads to the deduction about the spontaneous and abrupt interaction of the protein with its partner protein to perform efficient cellular signaling processes [39]. With the enhancement of the folding capability of the protein complex, it becomes more potent to perform its function [39]. The estimation for the free energy of folding for MDM2 protein before and after its interaction with DHFR was supported by the execution of VADAR 2.0 [39].

#### 2.11.2. Folding Rate of MDM2 Protein for Thermodynamic Stability

Thermodynamic steadiness in the sole protein (here, MDM2) remains a paramount feature to be calculated after the estimation of the free energy of folding calculation [41]. Folding rate evaluation of protein helps to investigate the thermodynamic steadiness in the MDM2 protein. In the process of evaluation, the time duration for the protein to undergo transformation is estimated [41]. SBpred module in the FDserver assisted in analyzing and examining the folding rate of the MDM2 protein before and after its interaction with DHFR protein [41].

#### 2.11.3. Net Area for Solvent Accessibility in the MDM2 Protein

For the investigation into the effectiveness of the interaction, net area obtainable for the solvent molecules to participate with the MDM2 protein was essential to be evaluated. It has been formerly documented that, upon stronger and firmer interaction, the value for net solvent accessible area obtainable for the solvent particles to participate with the protein gets reduced drastically [42]. So, the net vicinity obtainable for the solvent molecules to get access to the surface of the MDM2 protein before and after interaction from the simulated MDM2-DHFR complex was estimated to observe the potency in the interaction with DHFR protein.

### 2.12. Statistical Significances for the Evaluations

To rationalize and have an outlook for the calculated outcomes, statistical significances were evaluated for each and every estimated result. The statistical significance via data analysis was performed with the calculation of the paired *t*-test. In this test, the main accepted supposition is that the SD or standard deviation values should be unequal for both comparative parameters (here, the preinteractive MDM2 and postinteractive simulated MDM2). The outcomes are affirmed to be statistically significant when the *P* value from the respective *t*-tests is less than 5% or *P* < 0.05.

## 3. Results

### 3.1. Selection of the Best Suited Model after Comparative Study

Comparable study amongst the four variedly modeled MDM2 proteins from* Homo sapiens* had been performed based on the negativity in *Z*-score, percentage of residues that had a standard 3D-1D score ≥0.2 from the verify3D calculations, and the percentage of residues in the core (most favored) and disallowed regions from the Ramachandran plot. The comparison has been tabulated in Table 1. From the evaluations, the MDM2 model obtained by the execution of Phyre2 was observed to be the most stable one with the least *Z*-score value, 0% residues in the disallowed region, and maximum residual percentage to exhibit an average 3D-1D score ≥0.2. The template utilized for modeling the final selected protein model had a template ID: c2lzgA (fold library ID). The respective PDB ID was 2LZG with chain A. The PDB name of 2LZG_A is “NMR structure of mdm2 (6-125) with pip-1.” It possessed a confidence score of 100% and the % identity was also observed to be 100%.

### 3.2. Structural Level Demonstration of the Best Suited MDM2 Selected Protein

**Figure 1:**
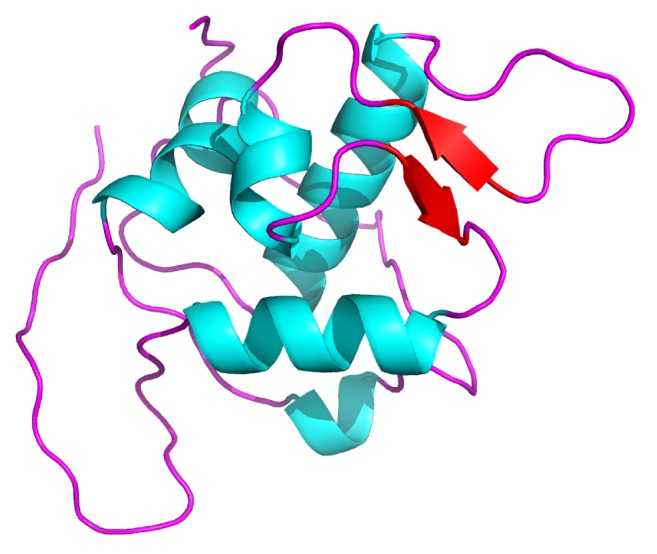
Structural and molecular view for the three-dimensional modeled functional MDM2 protein in* Homo sapiens*.

The satisfactorily 3D modeled MDM2 protein was observed to be 125 amino acid residues long. The protein had five sets of helical conformations, with two sets of *β*-sheets running antiparallel to each other, and these conformations are linked to one another with the help of coil-like conformations. The estimated percentage of residues forming helical and *β*-sheets results to be 32% and 8%, with the assistance of DSSP. The remaining 60% residues form coil-like conformations. The protein begins and ends with coils at the N- and C-terminal ends.  Figure 1 illustrates the detailed secondary structure conformation of the MDM2 protein from humans. The cyan shaded helices and red shaded *β*-sheets are seen to remain interconnected by magenta shaded coils in Figure 1.

### 3.3. Interactive Residues and Binding Patterns in the Final Refined and Simulated MDM2-DHFR Protein

**Figure 2:**
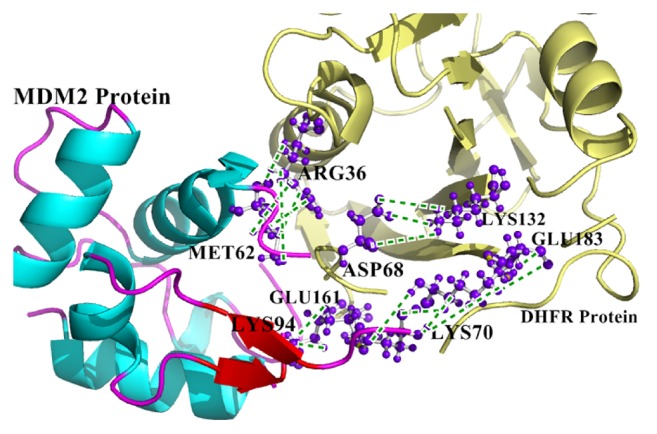
Few interactions in the final simulated and refined MDM2-DHFR protein complex in* Homo sapiens*. The residues are labelled with purple-blue “ball and stick” representation and the interactions are depicted in green dashed lines.

**Figure 3:**
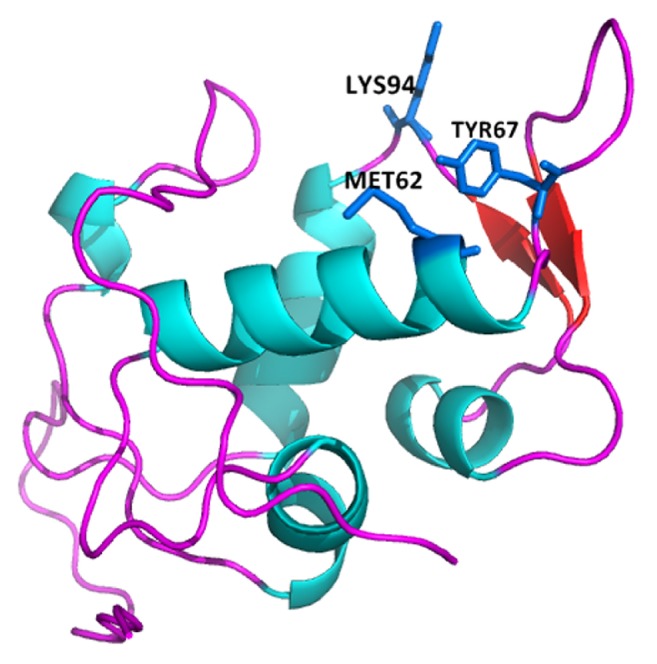
The essential paramount binding residues from MDM2 protein monomer that forms 50% of the interactions with DHFR protein.

Manifold interactive residues and their relevant positions from the DHFR and MDM2 protein in* Homo sapiens* were examined in detail. Among them, the prime important and essential ones for stabilizing the interaction were served by the side chain-side chain interactions and the ionic bonds. A net total of 18 side chain-side chain hydrogen bond interactions were observed to get accomplished between the final refined and simulated MDM2-DHFR protein complex (Table 2). There were many such residues that solely formed multiple interactions from their own specific positions. Moreover, surprisingly, efficient ionic interactions that are eminent in strengthening a protein-protein complex [35] were observed to be three in number (Table 3). Mainly, positively charged Lys residues from MDM2 formed the majority of the ionic bonds with negatively charged Glu residues from DHFR protein. An illustration for the protein-protein complex interaction has been demonstrated in Figure 2. The essential binding residues of MDM2 protein monomer (Met62, Tyr67, and Lys94) were found to form 50% of the interactions with DHFR protein (Figure 3). Therefore, this affirms stronger and stable participation of MDM2 and DHFR.

### 3.4. Evaluation from the Conformational Transitions in the MDM2 Protein (Preinteractive and Final Postinteractive)

**Figure 4:**
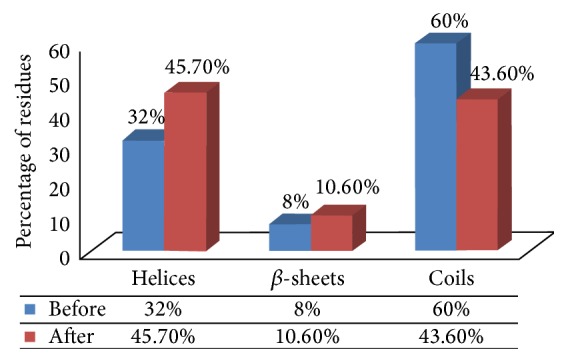
Transitions in the conformation of MDM2 protein from its preinteractive stage to its postinteractive stage from the simulated DHFR-MDM2 complex.

The conformational switches in the MDM2 protein before and after interaction from the final simulated MDM2-DHFR protein complex disclosed an abrupt transition in the secondary structural distribution in the MDM2 protein. The percentage of residues forming coil-like conformation got reduced from 60% to 43.60% in the final interactive structure of MDM2 (Figure 4). The increment in the percentage of residues adopting helical and *β*-sheets regions from 32% and 8% to 45.60% and 10.60%, respectively, additionally led to the disclosure of the strength in the backbone conformation of the protein (Figure 4). More than 50% of residues contributed to the coil-like conformation in the preinteractive protein, whereas more than 50% of residues contributed to the stronger helical and *β*-sheet-like conformation from the postinteractive MDM2 protein.

### 3.5. Stability Estimations in the MDM2 after Interaction with DHFR from the Final Simulated Complex

#### 3.5.1. Energy Changes for Preinteractive MDM2 Protein and MDM2-DHFR Complex

To investigate the effect upon the normal folding capability of the stable simulated MDM2-DHFR protein complex after their (MDM2 and DHFR protein) interaction, the free energy of folding value (in terms of kcal/mol) inferred a drastic increase in Δ*G* value from −107.23 kcal/mol to −218.7 kcal/mol (Table 4). This therefore deduces that the folding ability for the MDM2-DHFR complex got enhanced with a firmer and steady interaction for hampering the epigenetic regulation. Supportively, calculations from the DFire energy value also showed better steady interaction amongst MDM2 and DHFR proteins with an abrupt increment from −249.79 kcal/mol to −608.59 kcal/mol (Table 4). Therefore, altogether, this reveals the strong cooperative participation of MDM2 with DHFR protein from their (MDM2 and DHFR protein) final simulated stable complex.

#### 3.5.2. Rate of Folding per Second

To analyze the thermodynamic stability in the MDM2 protein after the estimation for free energy of folding estimation, the rate of folding per second for the MDM2 was evaluated and compared with the same from its preinteractive structure. The rate of folding per second in terms of log⁡(*K*_*f*_) was also analyzed to get altered from 1.43209/sec to 2.08699/sec after interaction (Table 4). This further affirms the stability of MDM2 after DHFR interaction.

#### 3.5.3. Net Area Obtainable for Solvent to Have Access upon the MDM2 Surface

The net area obtainable to the solvent from the surface residues from the MDM2 protein after interaction with DHFR was evaluated and compared with the net area available for solvent to achieve access to the residues from preinteractive MDM2 protein surface. The net solvent accessible area was also observed to get severely reduced from 7832.73 Å^2^ to 5675.83 Å^2^ after interaction (Table 4). This further implies the strong engagement of the residues in the interaction of DHFR for hampering of the nucleic acid metabolism and such cytological regulations.

### 3.6. Significance of the Outcomes via Statistical and Data Analysis

Each and every estimated upshot was analyzed to observe its statistical importance and thereby its validation. In every outcome, the interactive MDM2 protein from the final simulated complex of MDM2-DHFR was perceived to participate strongly with the DHFR protein. The increase in Δ*G* value for free energy of folding, DFire energy, and alteration in the log⁡(*K*_*f*_) value for folding rate per second was examined to exhibit a *P* value of 0.0011321, 0.0012413, and 0.0138213, respectively. On the other hand, the statistical evaluations for alteration in the net area obtainable for the solvent to interact with the MDM2 surface residues were observed to be significant with *P* = 0.02481562. The conformational transitions in the MDM2 also showed statistical significance holding a satisfactory *P* value of 0.01224914, 0.02319333, and 0.00231741 for the alteration in the percentage of residues adopting helical, *β*-sheets, and coil-like conformation after interaction with DHFR protein.

## 4. Discussion

This present* in silico* exploration focused on the participation of MDM2 protein and DHFR protein from* Homo sapiens*, and the alterations and transitions at the structural level for the MDM2 protein were disclosed. The past studies involving several wet laboratory experiments were undertaken for analyzing the degrading capability of MDM2 in several such proteins involved highly in the growth, proliferation, and signaling of the cells and tissues [5–7]. Even experiments involving yeast two-hybrid assays were performed for the disclosure of the fact that MDM2 protein interacts with DHFR to hamper the performance of DHFR protein and thereby hamper the DNA metabolism, synthesis of purines, and overall epigenetic regulation [7, 10]. Further, the prime necessity served by the folate metabolism gets obstructed [7]. So, this present study of the computational molecular analysis for the MDM2 protein in humans was examined.

For this purpose, the experimentally validated X-ray crystallographic structure of DHFR protein was obtained. For the interaction of DHFR with MDM2 protein, the human MDM2 protein sequence was selected and was modeled following varied discrete molecular modeling mechanisms or approaches. Among all the approaches and techniques followed to model the MDM2 protein satisfactorily, the structure generated with the operation of Phyre2 server served to be the most suited one in terms of all of its stereochemical features, mainly *Z*-scores from ProSA, percentage of residues having a mean 3D-1D score ≥0.2 from the verify3D estimations, and the residual distribution in the core and disallowed zones in the Ramachandran plot (Table 1). The 3D functional structure of the satisfactorily modeled MDM2 protein was therefore demonstrated at its structural level. Further, the two human proteins, DHFR and MDM2, were docked amongst themselves to study their interaction pattern and residual contribution from their respective positions. From the MD simulated and refined protein-protein complex, 18 side chain-side chain hydrogen bond interactions accompanied by 3 additional ionic interactions were observed to mainly strengthen the DHFR-MDM2 complex for the hindrance of cellular regulation and DNA metabolism (Tables 2 and 3). Lys94 and Lys70 from the MDM2 protein participated in forming ionic interactions with the two negatively charged glutamate residues (Glu161 and Glu183) from DHFR protein (Table 3). Further again, one lysine residue from the 132nd position in the DHFR protein interacted with Asp68 in the MDM2 protein to strengthen the interactions (Table 3). Other than these ionic interactions, 6 side chain-side chain interactions were solely formed by arginine residues in the DHFR protein from the 36th position (Table 2), among which 4 interactions were with Met62 and Tyr67 from the MDM2 protein. From DHFR protein, Asp168 formed four sole interactions with Gln18 and one interaction with the adjacent residue, Ser17, from the MDM2 protein (Table 2). Again, such kinds of bulk interactions were accomplished by Lys70 from MDM2 protein with 4 interactions with the DHFR protein and even Lys94 participated in the interaction from MDM2 (Table 2). Moreover, the binding residues in MDM2 monomer (Met62, Tyr67, and Lys94) were perceived to form 50% of the interactions with its partner DHFR protein (Figure 3). Altogether, due to these paramount interactions, a cavity in the DHFR protein by the positive environment of MDM2 protein thereby accommodates itself firmly to obstruct the cytological progressions.

Fascinatingly, to examine the strength in the interaction with DHFR and the alterations that MDM2 underwent after the interaction, several parameters were evaluated. It was observed that that MDM2 performed strong and firm interactions with DHFR with the severe ascent in the free energy of folding and DFire energy for the preinteractive MDM2 protein and postinteractive simulated DHFR-MDM2 protein complex. This affirms the better folding capability of the protein complex after interaction with an increased Δ*G* value. Furthermore, better folds are observed to be formed in the MDM2 protein after participation with DHFR protein from the alterations in folding rate. Severe reduction in the gross area obtainable for the solvent molecules to have access to the surface molecules of MDM2 also supports the compact and stable participation of DHFR protein with MDM2. Each and every outcome was examined to be statistically significant too, via the data analysis. On the whole, these deductions altogether were consensus to one another and apprehended the strong and active cooperation amongst the two human proteins, DHFR and MDM2, to cause a disturbance in the epigenetic regulation and folate metabolism, and therefore enhanced proliferation of the malignant cells.

Along with supportive statistical significances, additionally, an abrupt transition from coil-like conformation into mainly helices and *β*-sheets led to the disclosure of the steady conformation in the protein backbone upon its cooperative participation with DHFR protein from the simulated DHFR-MDM2 protein complex. The majority of the residues initially contributed to the coil-like conformation in the preinteractive MDM2 protein. On the other hand, upon interaction and simulation of the DHFR-MDM2 protein complex, the majority of residues in MDM2 protein contributed to predominantly helical conformations and *β*-sheets. Thus, it fortifies the proteins' backbone conformation. The overall study has been represented with a pictorial representation in Suppl. Figure 1 in Supplementary Material available online at http://dx.doi.org/10.1155/2016/9420692.

So far, in the preceding investigations, several such computational studies were investigated for the structural basis of varied proteins that are associated in varied human diseases [12, 13]. But, until now, no such computational or molecular level studies were performed to study the behavior of MDM2 protein from humans and its basic residual contribution when it participates with DHFR protein in humans, in a p53 (a paramount tumor suppressor protein) nonreliant manner. Therefore, captivatingly from this present study the basic molecular origin for hampering the epigenetic regulation, DNA, and nucleic acid pathways and obstructing the metabolism of folates and so on was studied and explored in detail. Therefore, further, this study instigates the future prospect for analyzing the mutational alterations at the molecular basis and thereby studying the interactive patterns with their alterations taking into consideration the wild-type proteins.

## 5. Conclusion and Future Scope

Folate metabolism and epigenetic regulation serve as one of the highly essential cytological phenomena in humans. DHFR protein plays a pivotal and unique role for the purpose, whereas human MDM2 often interacts with DHFR protein to hamper the sequential essential cytological processes. This leads to halting of apoptosis and thereby privileging of the progression of malignant cells. For the detailed molecular and structural basis exploration of the entire mechanism, in this current scenario, the alterations and transitions in the molecular and structural nature of the human MDM2 protein were investigated with an* in silico* outlook. It was performed by following discrete modeling techniques to model MDM2 protein in varied ways and, therefore, the best protein conformation was opted for after examining and comparing their stereochemical parameters. Optimization and energy minimization of the protein wereundertaken further to yield a stable and steady protein structure for MDM2. The three-dimensional functional structure of the MDM2 protein was thereby disclosed and this was succeeded by the analysis of the X-ray crystallographic structure of human DHFR protein for its interaction with MDM2 protein. The protein complex was stabilized to be analyzed in detail via MD simulations and refinements. Mainly, 18 side chain-side chain interactions and 3 strong ionic interactions were observed between the final simulated protein-protein complexes. Predominantly, lysine residues were observed to participate strongly with the strong interactions, thereby creating a pocket-like structure for the accommodation of MDM2 into the DHFR protein. Furthermore, the statistically significant disclosures for the increase in Δ*G* value for free energy of folding and DFire energy for the DHFR-MDM2 protein complex from the preinteractive stage of MDM2 protein apprehended their strong interaction by the enhancement in their folding capability. Fascinatingly, the alterations in the folding rate and net area for accessibility of solvent once again affirmed the stronger interaction by MDM2 with DHFR, thereby leading to the observed alterations in MDM2 itself. Additionally, the outcomes of statistically relevant conformational switches from chiefly coils to helices and sheets implied the stronger secondary structure conformation for MDM2 protein, thereby privileging the interaction with DHFR protein. These outcomes and observations would instigate the future research work for analyzing the mutational effects (whether beneficial or harmful) in either of the proteins and its (that is, the effects upon mutation) implications in the interaction pattern. This would further yield to prompt the investigations relating to the innovation of novel drugs, analyzing their interaction pattern and thereby serving as a prerogative for impelling the clinical research.

## Supplementary Material

The Supplementary Figure 1 shows the overall outcomes of the present study (that is, summing up of the entire results section or the outcomes of the study) in a tabular and flowchart representation.

## Figures and Tables

**Table 1:** Comparative analysis for the selection of the best model for MDM2 protein from *Homo sapiens*.

Parameters for the best model selection	MODELLER 9.14	Raptor-X	I-TASSER	Phyre2
Verify3D value	2.04%	48.45%	30.14%	69.60%
*Z*-score	−0.47	−3.62	−1.67	−5.32
Ramachandran plot				
Core	96.90%	88.90%	67.10%	90.20%
Disallowed	1.20%	3.80%	11.00%	0%

**Table 2:** Side chain-side chain hydrogen bond interactions in the final simulated and refined MDM2-DHFR protein complex in *Homo sapiens*.

Donor				Acceptor				Parameters	
Position	Protein	Residue	Atom	Position	Protein	Residue	Atom	Dd-a	Dh-a
36	D	ARG	NH1	62	M	MET	SD	3.64	4.1
36	D	ARG	NH1	62	M	MET	SD	3.64	3.73
36	D	ARG	NH2	62	M	MET	SD	3.57	3.98
36	D	ARG	NH2	62	M	MET	SD	3.57	3.69
36	D	ARG	NH1	67	M	TYR	OH	2.97	2.29
36	D	ARG	NH1	67	M	TYR	OH	2.97	2.91
162	D	TYR	OH	68	M	ASP	OD1	2.52	9.99
168	D	ASP	OD2	18	M	GLN	NE2	2.75	1.8
168	D	ASP	OD2	18	M	GLN	NE2	2.75	3
17	M	SER	OG	168	D	ASP	OD1	2.53	9.99
18	M	GLN	NE2	168	D	ASP	OD2	2.75	3.43
18	M	GLN	NE2	168	D	ASP	OD2	2.75	1.8
67	M	TYR	OH	37	D	MET	SD	3.31	9.99
70	M	LYS	NZ	130	D	HIS	NE2	3.35	9.99
70	M	LYS	NZ	183	D	GLU	OE1	2.66	9.99
70	M	LYS	NZ	183	D	GLU	OE2	2.8	9.99
70	M	LYS	NZ	185	D	ASN	OD1	2.76	9.99
94	M	LYS	NZ	161	D	GLU	OE2	2.63	9.99

Protein D and protein M represent DHFR and MDM2 proteins from *Homo sapiens*. Dd-a represents the distance between the “donor” and “acceptor.” Dh-a represents the distance between “hydrogen atom” and “acceptor.” The ATOM records, such as SD and NE, indicate the atomic names according to IUPAC (i.e., PDB) nomenclature as well as the CHARMM atom categories for each of the atoms in the individual residues. At the end of either of the ATOM records, the specific numbers (like NH1, OD2, etc.) represent the partial atomic charge.

**Table 3:** Ionic-ionic interactions in the final simulated and refined MDM2-DHFR protein complex in *Homo sapiens*.

Position	Residue	Protein	Position	Residue	Protein
132	LYS	D	68	ASP	M
161	GLU	D	94	LYS	M
183	GLU	D	70	LYS	M

Protein D and protein M represent DHFR and MDM2 proteins from *Homo sapiens*.

**Table 4:** Analysis for the strength in the interaction of MDM2 protein after interaction with DHFR protein/MDM2-DHFR complex.

Parameters for strength in interaction	Before interaction	After interaction
Free energy of folding (kcal/mol)	−107.23 (MDM2 protein)	−218.7 (protein complex)
DFire (kcal/mol)	−249.79 (MDM2 protein)	−608.59 (protein complex)
Folding rate for MDM2 protein	1.43209/sec	2.08699/sec
Net area for solvent accessibility for MDM2 protein	7832.73 Å^2^	5675.83 Å^2^

## Abbreviations

MD: Molecular dynamics
PIC: Protein Interaction Calculator
SD: Standard deviations
DHFR: Dihydrofolate reductase
MDM2: Mouse double minute 2.

## Additional Points

Highlights of the paper are the following: (i) varied molecular modeling techniques and stereochemical parameters, (ii) selection of the best suited protein model for obstructing cytological and epigenetic pathways, (iii) docking studies for residual disclosure for obstructing cytological and epigenetic pathways, (iv) stability and steadiness in interaction with supportive data significance, and (v) conformational switches with supportive data significance.
