# Supplementary material for: Structural Exploration and Conformational Transitions in MDM2 upon DHFR Interaction from Homo sapiens: A Computational Outlook for Malignancy via Epigenetic Disruption
Source: Scientifica (Cairo). 2016 Apr 17;2016:9420692. doi: 10.1155/2016/9420692 (PMC4860227; doi:10.1155/2016/9420692)
Supplement: Supplementary file 1 — The Supplementary Figure 1 shows the overall outcomes of the present study (that is, summing up of the entire results section or the outcomes of the study) in a tabular and flowchart representation. [file 9420692.f1.doc]

Supplementary Materials Legends

| **Supplementary Material Number** | **Supplementary Material Caption** |
| --- | --- |
| Suppl. Fig. 1 | Overall study for the entire work flow in a single pictorial representation |

**Supplementary Figure**


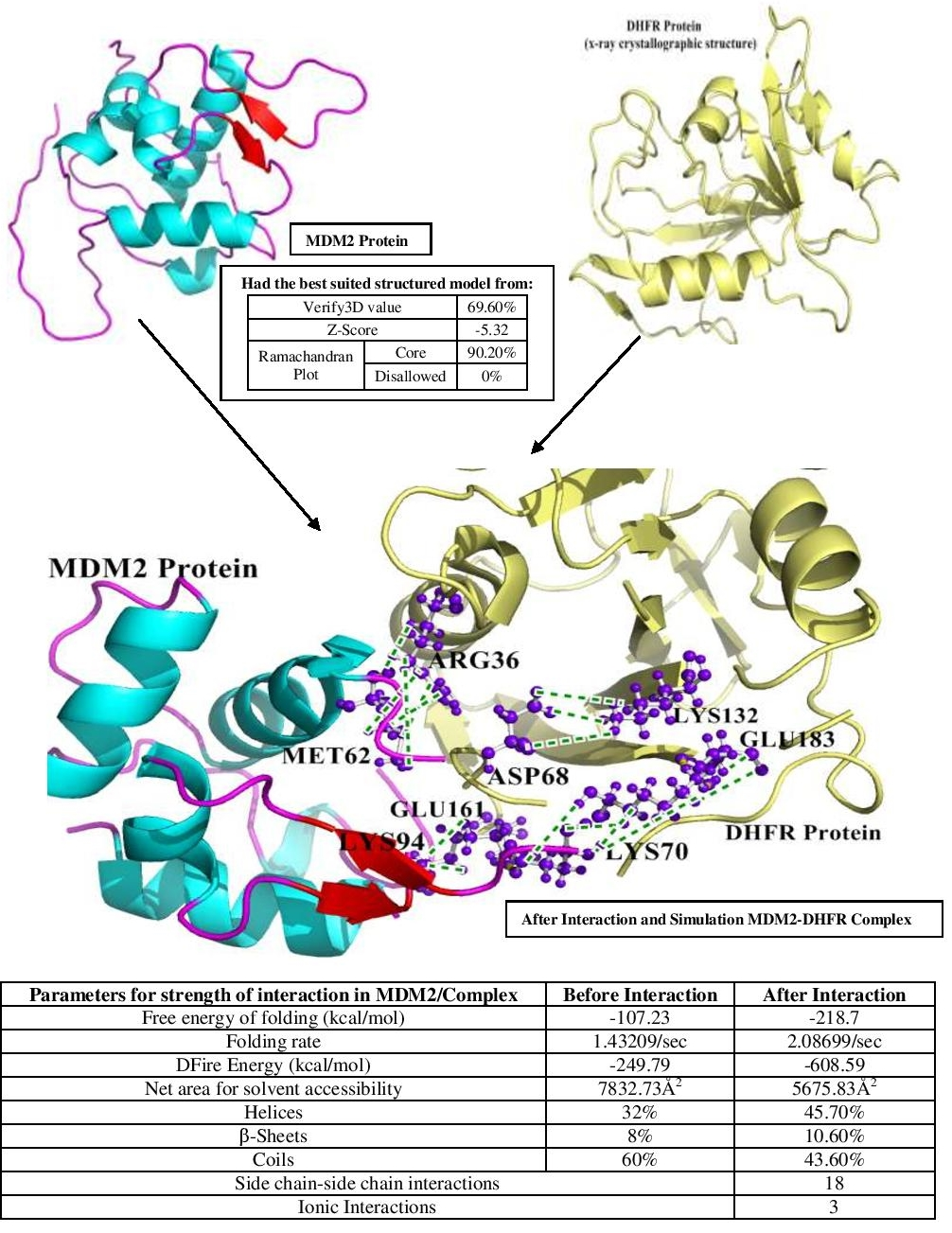


**Suppl. Fig. 1** Overall study for the entire work flow in a single pictorial representation
